# History of Febrile Caloricity, XLVII

**Published:** 1845-08

**Authors:** Bennet Dowler

**Affiliations:** New Orleans


### THE MEDICAL EXAMINER,

AND

### RECORD OF MEDICAL SCIENCE.

NEW SERIES—No. VIII.—AUGUST, 1845.

---

### ORIGINAL COMMUNICATIONS.

In a former number of the *Examiner*, (June, 1845,) we noticed some experiments on *febrile caloricity*, both before and after death, by Dr. Dowler. Since then, we have received from him the following additional observations, which we have much pleasure in laying before our readers. The subject is a most important one in its bearing on physiology, and we are pleased to find that Dr. D. is continuing his inquiries. It is only from multiplied and varied observations that we can feel assured of perfect accuracy.—EDITOR.

*History of Febrile Caloricity*, XLVII. (*Yellow Fever Dissection*, CXLIV.) By BENNET DOWLER, M. D., of New Orleans.

P. L., Irishman, aged 20; resident 9 months; a baker; large, muscular, and well proportioned; 4 days sick.

Aug. 15th, 4¾, P. M.; room 86°; hand 99°; axilla 104°.
16th, 4½, P. M.; room about 86°; hand 97°; axilla 104°.
18th, 2½, P. M.; room 90°; axilla 99°, nearly.
19th, 11¼, A. M.; room 90°; hand 97°; axilla 101°.
20th, died at 10¾, A. M., and was, in 15 minutes after, carried to the dead house, where the temperature was 86°; axilla 25 minutes after death 100°; 30 m., 105°; 35 m., 107°; 40 m., 107°; 45 m., 107°; 50 m., 107°; 55 m., 107°; 1 hour, 107°, nearly. Perineum, 1 hour and 10 minutes, 104°; 1 h. 15 m., 104°; 1 h.

20 m., 104°, nearly. Right epigast., 1 h. 25 m., 109° ; left, 1 h. 35 m. 108°; right iliac, 1 h. 45 m., 108° ; left, 1 h. 55 m., 108°; 2 h. 5 m., right epigast., 108° ; thigh, 2 h. 15 m., 109° ; 2 h. 25 m. 109°; heart and cardiac end of the gullet, 2 h. 35 m., 109° ; 2 h. 40 m., 109°; outer surface of the left lung, 2 h. 45 m., 106½°; mediastinum, 2 h. 50 m., 106½°; thigh, 2 h. 55 m., 106½°; 3 h. 106½°; right epigast., 3 h. 10 m., 106½°; left, 3 h. 15 m., 106½° ; heart, 3 h. 20 m. 106½° ; pelvic cavity, 3 h. 25 m., 106½°; thigh, 3 h. 30 m., 106½°; heart, 3 h. 35 m., 106½° ; thigh, 3 h. 45 m., 106½°. Being restricted in time, the observations were abandoned. Treatment—v. s., cups ; ol. ric. ; mercurials; sinap. ; enemata. The patient had copious nasal hæmorrhage, also much loss of blood from one of the many scarifications in cupping, towards the close of life.

*Hist.* XLV.—R. C., born in Kentucky, aged 25, resident 6 days; 22 hours before death,—hand in 10 m., 97½° ; axilla in 25 m., 104° ; air, 89°. At thirty minutes after death, (the body resting as usual on a stone floor without covering,) the experiments began. Every 10 to 20 minutes they were varied in the order following : axilla, 106½°; perineum and groin, 109°; rectum, 111° ; do., 111° ; thighs, without incisions, 102° ; rectum, 4 observations, each 109°. Axilla, after two hour's exposure, 100° ; rectum, 7 hours after death, 102°.

*Hist.* XLII.—(Dissection CXLVIII.) Miss M., resident nine months, aged 26 ; muscular and fat ; treated towards the close of her malady, (which, very slight at first, lasted 8 days,) with cal. 8 grs; ol. ricini ; sod. bicarb. ; morphia ; cinchon. ; serpent. ; porter ; beef tea. The experiments began at 30 minutes after death, and lasted more than three hours : from 40 minutes to 2 hours after death, the vagina gave 107°, without variation ; minimum of the axilla, at the first obs., 102° ; maximum, 105° ; more than an hour after death. The axilla then declined to 104°, and at 104° continued, without change, as long as watched, a period exceeding one hour. The vagina in the third hour declined to 105° ; the rectum to 104° ; the groin and axilla being the same. Air, 87°.

*Hist.* XVII. An Irishman, aged 36, last from Pass Christian, resident two weeks ; of gigantic frame, very muscular ; supposed to weigh 200 lbs ; gave in 19 minutes after death, 105° in the axilla ; in 26 m., over 106 ; and in 1 h. 26 m., nearly 105°. The thigh in 1 h. 34 m., 106° ; 1 h. 39 m., 107° ; 1 h. 44 m., 108°, still rising. Epigast., 1 h. 50 m., 106° ; brain, through the orbit, 1 h. 55 m., scarcely 101°. Air, 82°.

